# Tuberculous Trochanteritis: An Uncommon Presentation of Primary Tuberculosis Without Articular or Systemic Involvement

**DOI:** 10.7759/cureus.79201

**Published:** 2025-02-18

**Authors:** Samir Ben Salah, Mohammed Boubcher, Ayman Ben Abdellah, Nadir Miry, Najib Abdeljaouad

**Affiliations:** 1 Orthopedic Trauma Department, University Hospital Mohammed VI Oujda, Faculty of Medicine and Pharmacy of Oujda - Mohammed First University, Oujda, MAR; 2 Pathology Department, University Hospital Mohammed VI Oujda, Faculty of Medicine and Pharmacy of Oujda - Mohammed First University, Oujda, MAR; 3 Orthopedics Department, University Hospital Mohammed VI Oujda, Faculty of Medicine and Pharmacy of Oujda - Mohammed First University, Oujda, MAR

**Keywords:** hip joint, primary tuberculosis, trochanteritis, tuberculous, uncommon presentation

## Abstract

This paper reports the experience of our orthopedic department with a case involving a mass over the left greater trochanter that had been developing over nine months. Radiological evaluation (standard radiography + MRI of the left hip) revealed a soft tissue collection over the left greater trochanter with multiple calcifications originating from the trochanteric region. Management included surgical drainage of the collection, resection of calcifications, and collection of samples for bacteriological analysis (PCR for *Mycobacterium tuberculosis* DNA, which was negative) and histopathological examination. The histopathological findings confirmed a tuberculous infection, leading to the diagnosis of tuberculous trochanteritis.

## Introduction

Trochanteric tuberculosis of the hip is a rare condition (representing less than 2% of osteoarticular tuberculosis localizations) [1]. Its clinical presentation is nonspecific, as are the biological and radiological findings, making diagnosis challenging and often leading to confusion with other pathologies [1]. Though biological investigations may reveal an inflammatory syndrome (elevated erythrocyte sedimentation rate (ESR) and c-reactive protein (CRP)), they do not confirm the diagnosis. Similarly, imaging examinations (X-rays, MRI, CT scan) can show indirect signs such as soft tissue edema, bone erosions, or abscesses, but these abnormalities are not specific to tuberculosis [2]. As a result, the diagnosis is often delayed, and trochanteric tuberculosis is frequently identified at an advanced stage, marked by complications such as abscess or fistula formation with histological confirmation [2]. Therapeutic options must first address complications if they exist, and in the absence of complications, treatment is medical, based on antituberculous therapy, while surgery is primarily reserved for complications [3].

## Case presentation

**Figure 1:**
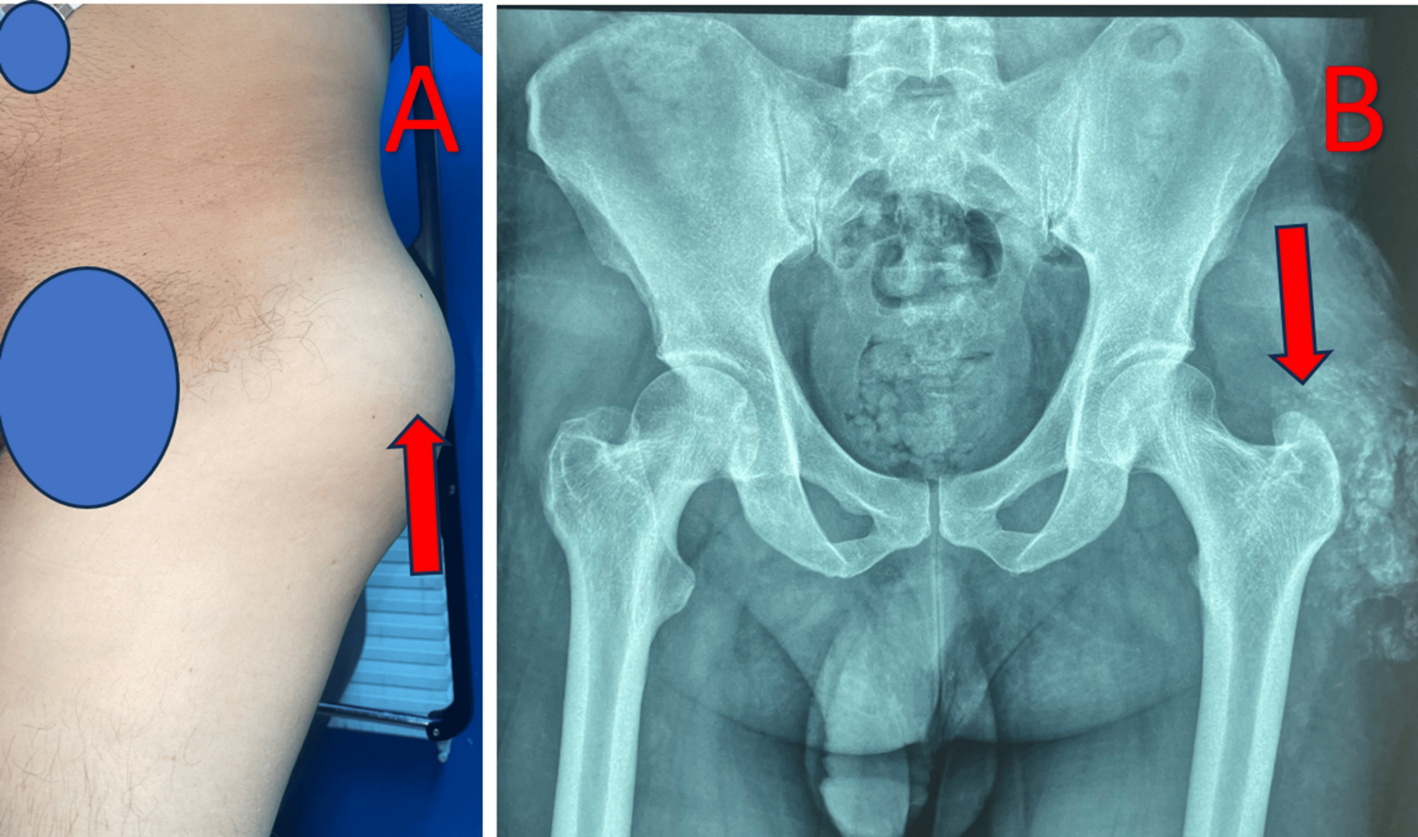
Clinical and radiographic presentation of the swelling A: Clinical appearance of the mass over the left greater trochanter (arrow). B: Standard pelvic radiograph showing calcifications over the left greater trochanter (arrow).

**Figure 2:**
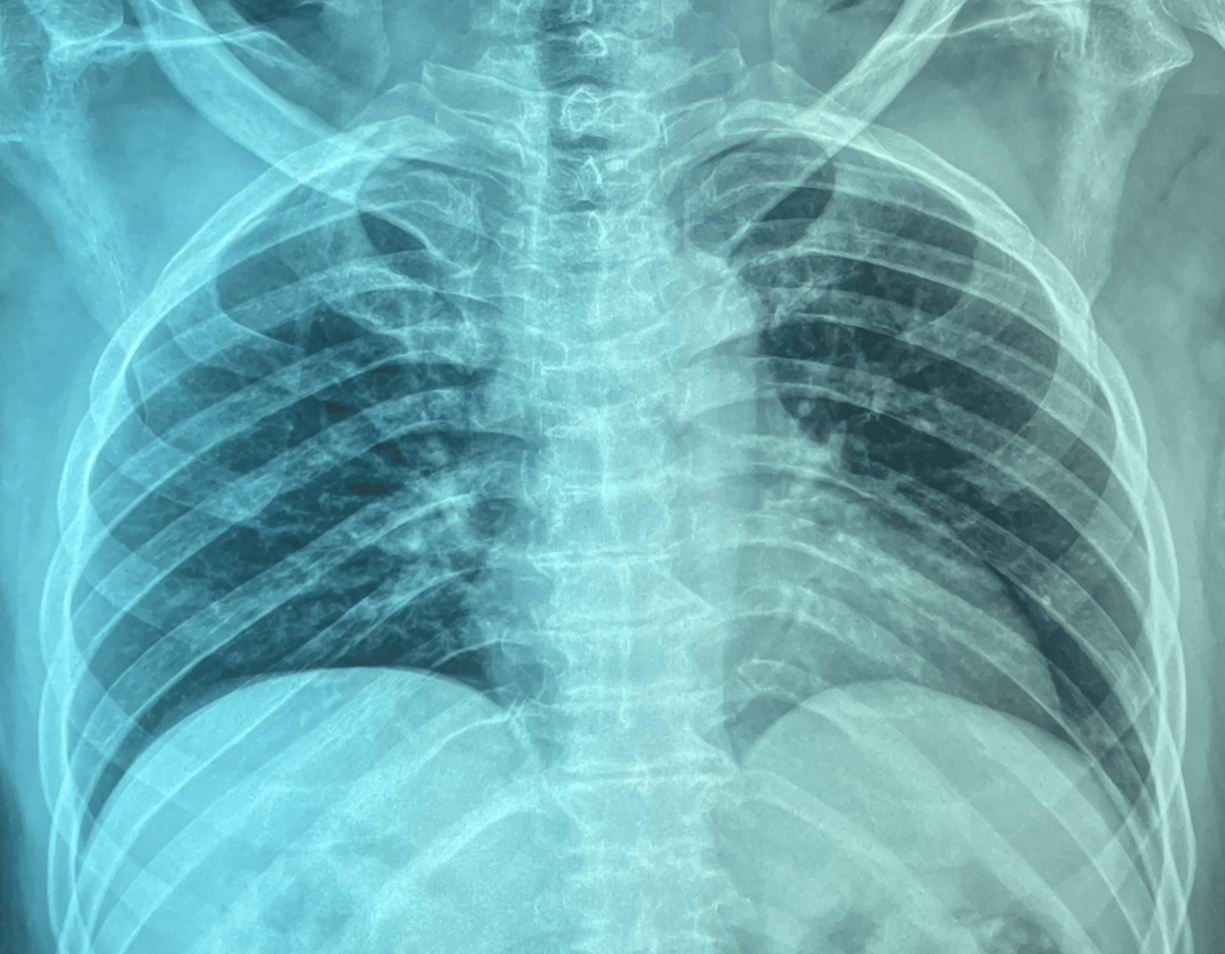
Normal chest X-ray

A 47-year-old male patient with no significant medical history presented with a swelling on the lateral aspect of the left hip, which had been present for nine months, accompanied by a fever of 38.5°C without other associated symptoms. Physical examination revealed a conscious patient with stable hemodynamic and respiratory status with a tender, non-fistulating mass over the left greater trochanter. Laboratory tests showed elevated inflammatory markers (CRP at 167 and white blood cell count). Standard pelvic radiography revealed calcifications over the left greater trochanter extending into the adjacent soft tissues, with no articular abnormalities. A chest X-ray was normal (Figures 1-2). 

**Figure 3:**
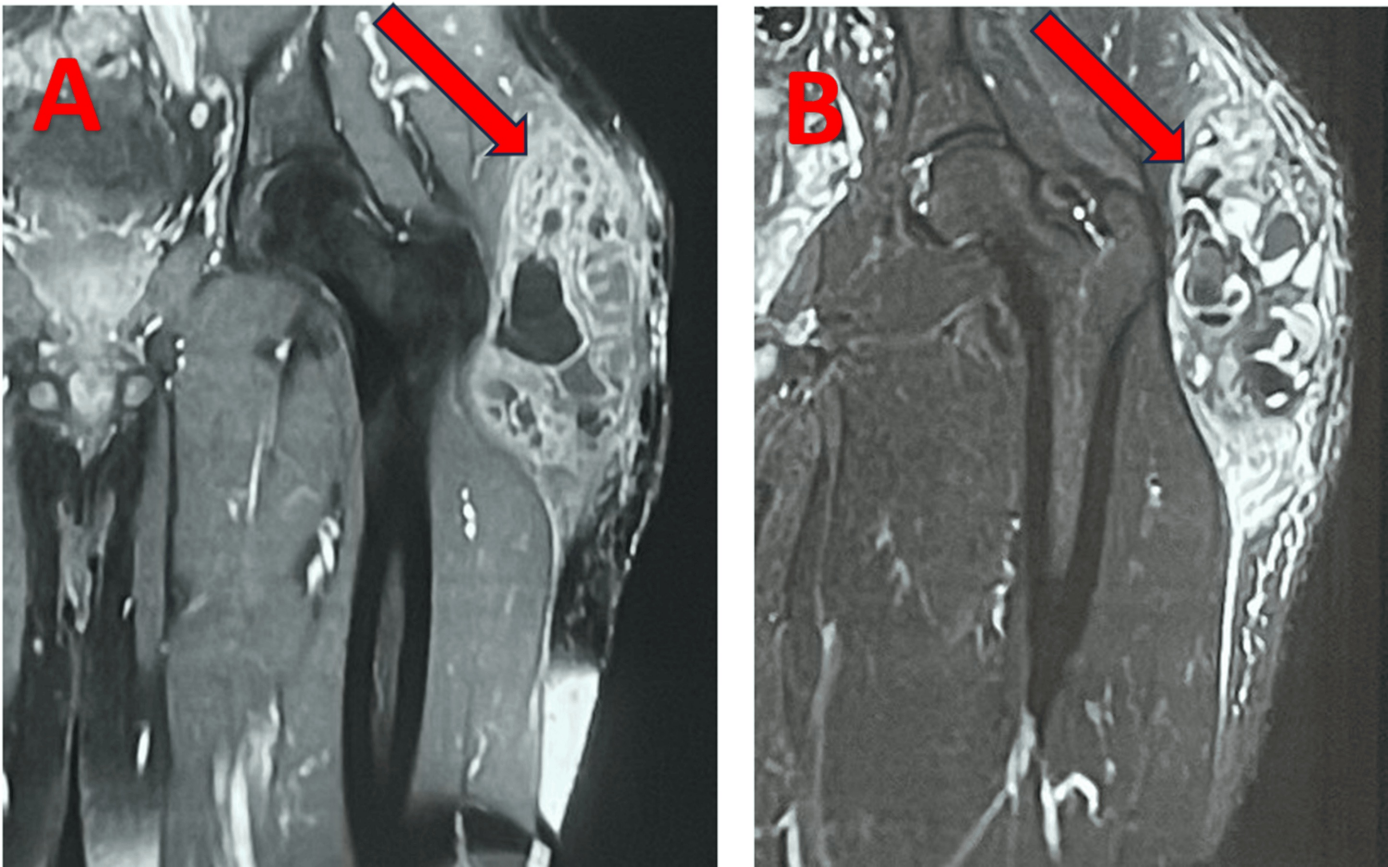
MRI findings of the left hip: A: Non-contrast MRI (coronal view) showing a collection with multiple calcifications over the left greater trochanter (arrow). B: Contrast-enhanced MRI (coronal view) showing peripheral enhancement of the collection (arrow).

**Figure 4:**
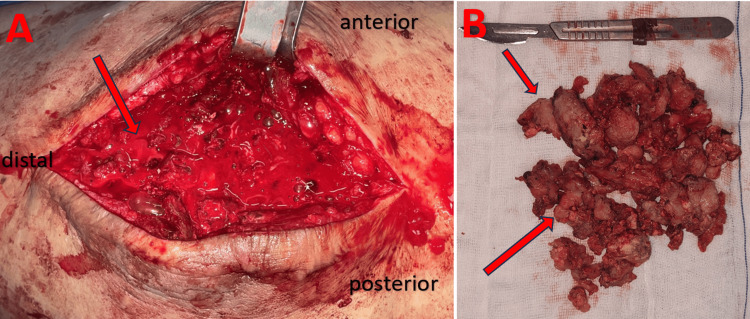
Intraoperative findings: A: Immediate appearance after incision of the collection (arrow). B: Appearance of the resected calcifications (arrows).

MRI of the left hip with and without contrast confirmed the presence of a soft tissue collection over the left greater trochanter with multiple calcifications and no signs of malignancy (Figure 3). Surgical management included drainage of the collection and resection of calcifications under general anesthesia. The presence of frank pus prompted the collection of five samples for cytobacteriological analysis, including tuberculosis testing. Biopsies of the calcifications, greater trochanter, and soft tissues were taken for histopathological examination (Figure 4). Serology for brucellosis was negative, and PCR for *Mycobacterium tuberculosis* complex DNA was also negative.

**Figure 5:**
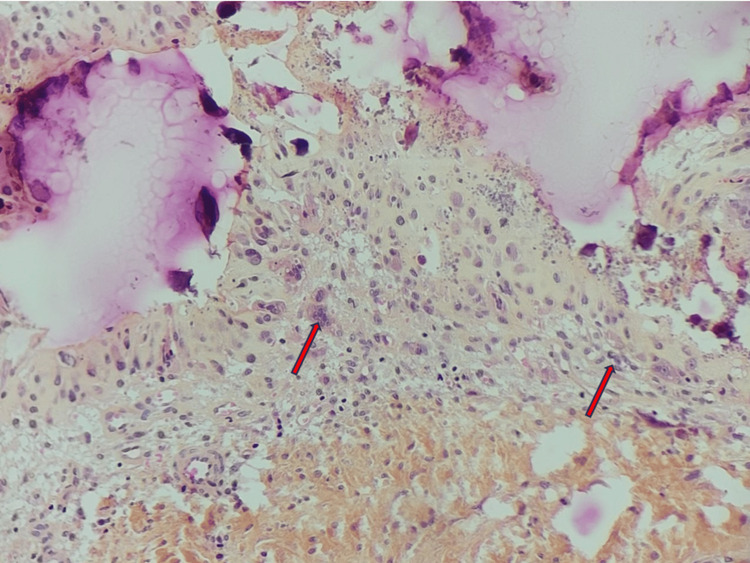
Photomicrograph of the biopsy reveals a granulomatous reaction composed of multiple epithelioid cells and multinucleated giant cells (arrows) (HES, x200). HES: Hematoxylin, eosin, and saffron.

Histopathological examination of the biopsies confirmed the diagnosis of tuberculosis (Figure 5). The patient was started on a nine-month antitubercular treatment regimen in accordance with the Moroccan National Tuberculosis Control Program, resulting in a favorable outcome [4].

## Discussion

Tuberculous infection localized to the greater trochanter is a rare occurrence, accounting for less than 2% of cases in individuals with known tuberculous infections [3]. The presence of primary tuberculous trochanteritis, without evidence of infection elsewhere or a prior history of tuberculosis, makes this case exceptionally rare and clinically significant. The clinical presentation of tuberculous trochanteritis is often nonspecific and insidious, evolving over an extended period, which complicates the diagnostic process. In most cases, the diagnosis is made only after the development of complications such as abscess formation or fistulization, as was observed in our patient, who sought medical attention only after the onset of pain and fever [5].

The diagnosis of tuberculous trochanteritis must be confirmed through bacteriological and/or histopathological examination [6]. In our case, *Mycobacterium tuberculosis* was not isolated, while epithelioid and giant cell granulomas were identified. In the absence of microbiological confirmation, the diagnosis relies on epidemiological, clinical, and evolutionary criteria.

The cornerstone of treatment is specific antitubercular therapy, which typically involves a four-drug regimen for the initial two months, followed by a continuation phase with isoniazid and rifampicin. The total duration of treatment may range from 10 to 18 months, depending on the extent and severity of the lesions [7].

In our case, PCR testing for *Mycobacterium tuberculosis* DNA was negative, reinforcing the findings of previous studies that a negative PCR result does not exclude tuberculous infection. Histopathological evaluation remains essential for definitive diagnosis [8]. The rarity of primary tuberculous trochanteritis, combined with the diagnostic challenges posed by its nonspecific presentation, makes this case particularly noteworthy and worthy of discussion and dissemination within the medical community [9]. Primary tuberculous trochanteritis is an exceptionally rare condition that requires a high index of suspicion, particularly in patients without a history of tuberculosis or evidence of systemic infection. The insidious nature of the disease and the potential for delayed diagnosis underscore the importance of histopathological confirmation. This case highlights the need for clinicians to consider tuberculous trochanteritis in the differential diagnosis of chronic hip pain or swelling, even in the absence of systemic symptoms or a positive microbiological test. Early recognition and appropriate treatment are crucial for achieving favorable outcomes.

## Conclusions

Tuberculous trochanteritis of the trochanteric region, although rare, should not be ruled out, even in patients with no history of tuberculosis or other tuberculous diseases. This condition can present silently and progress slowly over an extended period, particularly in endemic regions. Patients may exhibit subtle symptoms, making diagnosis challenging. The significance of this exceptional case lies in the fact that tuberculous trochanteritis can be a primary form of infection. Considering this possibility enables earlier detection and appropriate management, thereby preventing severe complications such as abscesses and fistulas. Increased vigilance and clinical suspicion are therefore essential to avoid delays in diagnosing and treating this rare but significant condition.
